# Soil Matrix Determines the Outcome of Interaction Between Mycorrhizal Symbiosis and Biochar for *Andropogon gerardii* Growth and Nutrition

**DOI:** 10.3389/fmicb.2018.02862

**Published:** 2018-11-27

**Authors:** Zahra Paymaneh, Milan Gryndler, Tereza Konvalinková, Oldřich Benada, Jan Borovička, Petra Bukovská, David Püschel, Veronika Řezáčová, Mehdi Sarcheshmehpour, Jan Jansa

**Affiliations:** ^1^Department of Soil Science, Faculty of Agriculture, Shahid Bahonar University of Kerman, Kerman, Iran; ^2^Laboratory of Fungal Biology, Institute of Microbiology, Czech Academy of Sciences, Prague, Czechia; ^3^Faculty of Science, Jan Evangelista Purkyně University in Ústí nad Labem, Ústí nad Labem, Czechia; ^4^Laboratory of Molecular Structure Characterization, Institute of Microbiology, Czech Academy of Sciences, Prague, Czechia; ^5^Institute of Geology, Czech Academy of Sciences, Prague, Czechia; ^6^Institute of Botany, Czech Academy of Sciences, Průhonice, Czechia

**Keywords:** arbuscular mycorrhizal fungi, community, historic biochar, mycorrhizal response, nitrogen, phosphorus

## Abstract

Biochar has been heralded as a multipurpose soil amendment to sustainably increase soil fertility and crop yields, affect soil hydraulic properties, reduce nutrient losses, and sequester carbon. Some of the most spectacular results of biochar (and organic nutrient) inputs are the *terra preta* soils in the Amazon, dark anthropogenic soils with extremely high fertility sustained over centuries. Such soil improvements have been particularly difficult to achieve on a short run, leading to speculations that biochar may need to age (weather) in soil to show its best. Further, interaction of biochar with arbuscular mycorrhizal fungi (AMF), important root symbionts of a great majority of terrestrial plants including most agricultural crops, remains little explored. To study the effect of aged biochar on highly mycotrophic *Andropogon gerardii* plants and their associated AMF, we made use of softwood biochar, collected from a historic charcoal burning site. This biochar (either untreated or chemically activated, the latter serving as a proxy for freshly prepared biochar) was added into two agricultural soils (acid or alkaline), and compared to soils without biochar. These treatments were further crossed with inoculation with a synthetic AMF community to address possible interactions between biochar and the AMF. Biochar application was generally detrimental for growth and mineral nutrition of our experimental plants, but had no effect on the extent of their root colonized by the AMF, nor did it affect composition of their root-borne AMF communities. In contrast, biochar affected development of two out of five AMF (*Claroideoglomus* and *Funneliformis*) in the soil. Establishment of symbiosis with AMF largely mitigated biochar-induced suppression of plant growth and mineral nutrition, mainly by improving plant acquisition of phosphorus. Both mycorrhizal and non-mycorrhizal plants grew well in the acid soil without biochar application, whereas non-mycorrhizal plants remained stunted in the alkaline soils under all situations (with or without biochar). These different and strong effects indicate that response of plants to biochar application are largely dependent on soil matrix and also on microbes such as AMF, and call for further research to enable qualified predictions of the effects of different biochar applications on field-grown crops and soil processes.

## Introduction

Biochar (*sensu lato*, including charcoal and activated biochar) is a solid product of exposure of any organic (carbonaceous) materials such as plant biomass (be it wood, grass biomass or straw) or different kinds of organic waste matter to heat under total or partial absence of oxygen (Hagemann et al., 2018). It is formed either during wildfires or intentionally produced to obtain charcoal/biochar to be used as a fuel or for industrial application (e.g., sorbent) or for soil amendments. It contains highly condensed carbon (C) and by-products of the charring process such as bio-oils and tars, including polycyclic aromatic hydrocarbons (PAHs, which are particularly abundant in fresh biochar, Zhu et al., 2017 and references therein). Application of biochar to soils has previously been proposed to substantially and sustainably increase soil fertility, water holding capacity, and also to sequester C as a mitigation measure to offset anthropogenic CO_2_ emissions (Atkinson et al., 2010; Roberts et al., 2010). These suggestions originate from existence of *terra preta* soils in the Amazon and elsewhere in the tropics, where generally unfertile, highly weathered and mostly acid soils have historically been managed by biochar and organic nutrient additions to improve crop nutrition and yields (Glaser and Birk, 2012; Mao et al., 2012). Proposed mechanisms of such soil improvements were enhanced nutrient, mainly nitrogen (N) and phosphorus (P) availabilities, and limitation of nutrient (particularly N) losses to the environment, remediation of pH extremes (particularly the low pH of some of the tropical soils), reducing toxicity of metals (such as aluminum and manganese) and improving cation exchange capacity of the soils (Schulz and Glaser, 2012; Alling et al., 2014; Gul and Whalen, 2016). Further, biochar amendments can improve water holding capacity in sandy soils and aeration in heavy clays through affecting soil porosity (Mickan et al., 2016; Koide, 2017). In contrast to tropical soils, where biochar applications sometimes lead to truly spectacular effects, applications of biochar to temperate soils has generally caused much weaker effects on plants and on the soil quality (Borchard et al., 2012; Jeffery et al., 2017; Koide, 2017). Previously, this has partly been attributed to the fact that biochar was applied fresh in most studies with temperate soils. And it has also been suggested that greatest benefits would only be achieved when biochar slowly weathers and interacts with minerals in the soil over large temporal scales (Atkinson et al., 2010; Borchard et al., 2012; Ren et al., 2018). Such long-term phenomena are, however, very difficult to study directly. Further, it has been proposed that only certain types of biochar could effectively ameliorate certain soil properties such as pH extremes, textural limitations and bioavailability of toxic metals, depending on the biochar feedstock and also on the charring conditions such as temperature and oxygen availability (Jeffery et al., 2011; Kloss et al., 2012; Butnan et al., 2015). In consequence, the type of biochar and also its application rates may need to be finely tuned up for each recipient soil/ecosystem, so as to fit the plant, environment, nutrient inputs and forms, as well as abiotic and biotic soil conditions (Lehmann et al., 2011; Shen et al., 2016; Luo et al., 2017). Particularly, the interactions of biochar with soil microbes including the arbuscular mycorrhizal fungi (AMF) received relatively little experimental attention so far, although evidence for such interactions is currently mounting from different experimental systems (Mickan et al., 2016; Luo et al., 2017; Ohsowski et al., 2018). For example, the ratio between AMF and saprotrophic soil fungal abundances in soil increased due to high rates (30 and 50 tons ha^-1^) of field biochar application and the saprotrophic fungal to bacterial abundance ratio increased in the 50 tons biochar ha^-1^ treatment in northwest China (Luo et al., 2017). Recent meta-analysis synthesizing effects of biochar on soil microbial communities (Zhang et al., 2018) largely confirmed that biochar soil amendments generally increased soil fungal to bacterial biomass ratios, ratios of Gram-positive to Gram-negative bacteria, as well as total microbial biomass and activity. Biochar also significantly modulated dynamics of ammonia oxidizers in soil, increased their abundance and caused shift in their community composition (promoting diversity of ammonia oxidizing bacteria in contrast to ammonia oxidizing archaea, Song et al., 2014). Different mechanisms behind these effects were suggested such as promotion of soil aggregate formation due to significant inputs of labile C and N with biochar application (particularly of the low-temperature pyrolyzed biochar), shifting soil pH (low-temperature pyrolysis yields biochar with lower pH than higher pyrolysis temperatures), inputs of toxic/signaling compounds with biochar such as PAHs and flavonoids, which could directly affect soil microbes, and also modulating soil properties such as cation exchange capacity and water availability and formation of organo-mineral layers over time (Zhang et al., 2018 and references therein). Biochar has also been proposed to protect beneficial soil microbes (particularly the bacteria) from their predators by providing refugia of appropriate sizes (Lehmann et al., 2011).

Particularly important could be the effects of biochar on the AMF because of their tight functional linkages to the plants. The AMF establish symbiotic relationship with roots of more than a half of extant plant species including many important crop and grassland species. This symbiosis is responsible for up to 100% of phosphorus (P) uptake of the mycorrhizal plants and also for a significant share of nitrogen (N) uptake by the plants, particularly from the organic N sources (Hodge et al., 2001; Smith et al., 2004; van der Heijden et al., 2008; Hodge and Storer, 2015). It has been reported that AMF hyphae could penetrate biochar particles and gain P that has transiently been adsorbed onto their surfaces (Hammer et al., 2014). Further, biochar amendment to soil exposed to drought promoted the growth of AMF extraradical hyphae in such soils as compared to unamended soil (Mickan et al., 2016). Analysis of microbial communities in African Dark Earths indicated absence of a strong effect of biochar on indigenous AMF (while reporting significantly higher fungal to bacterial ratio) in spite of elevated mineral fertility of such soils as compared to unamended soils (Camenzind et al., 2018). This absence of strong effect of biochar on mycorrhizal abundance is also consistent with the message conveyed from earlier meta-analysis by Biederman and Harpole (2013). However, biochar could still indirectly affect the AMF community and its functioning through changing abiotic soil properties such as pH (Hazard et al., 2013; Jansa et al., 2014), modulating the composition and activity of microbial communities in soil (Luo et al., 2017), and/or affecting biological soil processes such as nitrification (Schulz and Glaser, 2012; Prommer et al., 2014; Gul and Whalen, 2016) – because these processes could importantly feed back on the AMF functioning (e.g., Cheng et al., 2012; Bukovská et al., 2018).

The aim of this study was thus to address interactions between biochar and mycorrhiza with respect to growth and mineral (P and N) nutrition of a highly mycotrophic host plant *Andropogon gerardii* (Püschel et al., 2016; Bukovská et al., 2018), in two different temperate agricultural soils – one developed on a granitic moraine (acid), and the other derived from calcareous river sediments (alkaline). Instead of using freshly prepared biochar, we used historic biochar that has been “composted” for a number of decades in a forest soil, so as to better mimic processes that would only establish after some time from biochar addition to the soil. We used this biochar either untreated or activated through autoclaving and treating it with hydrogen peroxide. The latter treatment has been included as a proxy for freshly prepared biochar, where the biochar active surfaces and pores are typically devoid of microbes or products of their activity and usually show different physico-chemical properties (e.g., greater cation sorption capacity) than aged biochar (Zhu et al., 2017 and references therein). We expected additive and positive effects of both biochar and mycorrhiza on plant P nutrition (in agreement with previous studies, Hammer et al., 2015; Liu et al., 2018). Further, we also expected negative interactions between N nutrition of plants and biochar amendment, based on current literature demonstrating slowing down some pathways involved in soil N cycling due to biochar application (Prommer et al., 2014). To address this latter point, we provided isotopically (^15^N-) labeled organic N source in a spatially discrete soil patch beyond direct reach of the roots but accessible to AMF hyphae (similarly as in Řezáčová et al., 2018), and measured N transfer to plants by using ^15^N-isotopic analyses of the plant tissues. Besides, we also addressed whether soil properties and/or biochar amendment affected composition of synthetic AMF communities, by using previously developed quantitative real-time PCR protocol (Thonar et al., 2012).

## Materials and Methods

### Experimental Design

The experiment included all combinations of the three following factor levels: (1) Soil (either acid or alkaline), (2) Mycorrhizal inoculation (with or without an infective AMF community), and (3) Amendment with biochar (none, native historic biochar, or the same biochar activated by autoclaving and hydrogen peroxide). The experiment was carried out in 1-l pots under glasshouse conditions in a completely randomized design with five replicate pots per each of the 12 treatments (i.e., 60 pots altogether).

### Pot Experiment Setup

The pots were filled with soil mixed or not with biochar (3.6% by weight, 200 g in total of 5,500 g soil prepared per each treatment) and the mycorrhizal inoculum, alive or autoclaved (9.1% by weight, 500 g in total of 5,500 g soil prepared per each treatment). Further, each pot was supplied with a small root-free patch [a plastic tube with a diameter of 3.6 cm and 3 cm long, covered at both openings with a hydrophilic 40-μm root exclusion mesh manufactured from polyamide (Silk & Progress, Brněnec, Czechia)]. The mesh excluded roots, but allowed most microbes including fungi as well as aqueous solutions to move through the pores. The root-free patches were buried at a depth of 6 cm below surface before sowing the plants. The patches were filled with 41 g the same material as the rest of each of the pots, and supplemented with organic N source (^15^N-labeled clover biomass, described in Bukovská et al., 2018; 205 mg per patch, i.e., 0.5% enrichment by weight). Microbial wash from a previous pot cultures grown under the same conditions as the pots used for production of mycorrhizal inoculum but lacking any AMF propagules (i.e., non-mycorrhizal inoculum pots, also called mock inoculum) was added to all pots to equalize composition of microbial communities at the beginning of the experiment (Gryndler et al., 2018). To this end, potting substrate from the non-mycorrhizal inoculum pots was mixed with water in proportion 1:10 (*w:v*) and filtered through 40-μm analytical sieve. This suspension was then added and mixed to all soils at a rate of 200 ml per 5,500 g of soil (i.e., 3.6% *v:w*).

### Biochar

**FIGURE 1:**
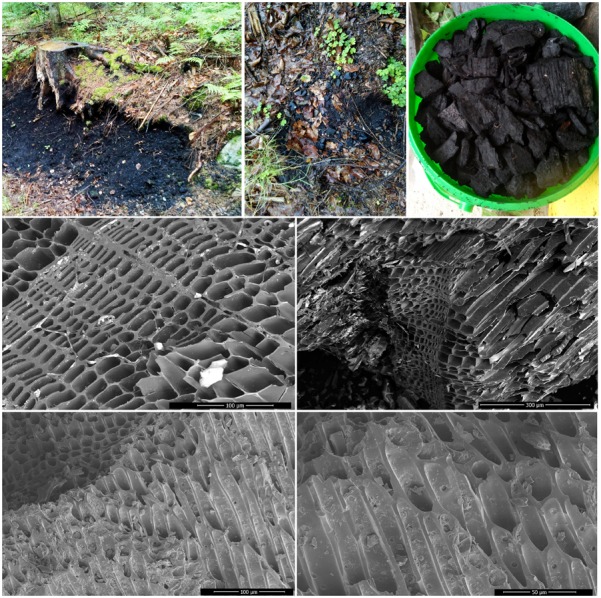
Remnants of a historic charcoal pile under a tree stump and a photo of charcoal fragments recovered from that site (upper row). Scanning electron microphotographs of the biochar recovered from the historic charcoal burning site (native biochar, middle row) and of the biochar activated by autoclaving and hydrogen peroxide treatment (bottom row).

The native softwood biochar was obtained from a place where charcoal was historically produced for industry applications (glassworks) using large charcoal piles, and was at least 70 years old, deposited under forest litter, overgrown by trees (Figure 1), and sparsely colonized by fungal hyphae (Figure 1). It was collected in large pieces (2–7 cm in diameter), broken to small particles by a hammer and sieved through a 2-mm sieve. It was used either untreated (unsterile, native) or activated in two steps: (1) Autoclaving at 121°C for 30 min, cooled down and incubated at room temperature for 7 days. (2) Subjected to oxidation by 10% H_2_O_2_ (3 l of such hydrogen peroxide solution added to 1 kg of the biochar). After 30 min from addition of the peroxide, the temperature of the slurry raised to +80°C, at which point we added 3 l of distilled water and cooled the slurry in snow (0°C). The solution was then separated from the biochar by filtration through glass fiber paper. Initial eluent was black–brown. The biochar was then thoroughly washed with 1 l of distilled water three times until the eluent was only slightly colored, and dried in the oven at 65°C for 2 days, yielding about 840 g of dry activated biochar (starting with 1 kg of native biochar).

### Soils, Plants, and the Glasshouse

Two kinds of soil were used in the experiment described here: Acid soil was collected from Tänikon, Switzerland (pH = 6.5, for further details please see Table 1 and Jansa et al., 2003), while alkaline soil was collected in Litoměřice, Czechia (pH = 7.8, for further details please see Table 1 and Řezáčová et al., 2016). Both soils were air-dried at room temperature and sieved (<8 mm), homogenized and sterilized by gamma-irradiation (min 25 kGy) 3 months prior to the pot experiment described here. The pots were sown with *Andropogon gerardii* (30 seeds per pot) provided by Jelitto Staudensamen GmbH (Schwarmstedt, Germany), and the plants were grown in experimental glasshouse of the Institute of Microbiology in Prague under supplemental lighting providing a minimum of 150 μmol m^-2^ s^-1^ photosynthetically active radiation over a 14 h photoperiod. Temperature in the glasshouse fluctuated between 20°C and 30°C during the experiment, with some few warmer episodes (see temperature log in the Supplementary Table S1). Pots were watered daily with deionized water to maintain approximately 80% water holding capacity of the soils. No fertilization was provided with the irrigation water throughout the experiment. Yellow charts were installed around the pots to catch adult sciarid flies (*Bradysia paupera*) throughout the cultivation, and, additionally, a pyrethroid insecticide (Karate, Syngenta) was sprayed twice to all pots throughout the cultivation. A few pots, which had most of the plants dead due to the activity of the flies, were re-sown with 30 seeds of *Andropogon gerardii* during the first 3 weeks of the experiment. The duration of the cultivation was 65 days altogether.

**Table 1:** Chemical properties of the differently amended soils and the biochars before cultivation of the plants.

Material	Amendment	pH	P total ^1^ (mg/kg)	P water extractable ^2^ (mg/kg)	P immediately available ^3^ (mg/kg)	C (%)	N (%)
Acid soil	None	6.53	614	4.31	2.45	1.99	0.11
Acid soil	Active biochar	6.49	579	1.57	2.14	3.76	0.14
Acid soil	Native biochar	6.84	772	1.35	1.64	3.58	0.14
Alkaline soil	None	7.80	658	4.14	2.45	0.79	0.08
Alkaline soil	Active biochar	7.13	539	4.17	0.72	2.77	0.13
Alkaline soil	Native biochar	7.08	588	3.22	0.46	3.86	0.18
Biochar active		3.46	133	38.8	38.1	51.2	0.30
Biochar native		4.36	80.8	6.86	3.97	57.0	0.31

*Phosphorus (P) concentrations were analyzed spectrophotometrically at 610 nm according to Ohno and Zibilske (1991), and the concentrations of total organic carbon (C) and total organic nitrogen (N) were measured using Flash 2000 elemental analyzer (Thermo Fisher Scientific). Average values out of two technical replicates each are shown. ^*1*^ P concentration in the soils/biochar measured after incineration of the samples at 550°C for 12 h and extraction of the ashes with concentrated (65%) and hot (200°C) HNO_*3*_. ^*2*^ P concentration in the soils/biochar measured in water extracts (1:10, w:v) after shaking for 18 h and filtration through 0.22 μm membrane filter. ^*3*^ Concentration of instantaneously available P in the samples (exchangeable within 1 min of isotopic exchange), assessed by the Isotope Exchange Kinetics method and carried out according to Frossard and Sinaj (1998)*.

### Mycorrhizal Fungi

Synthetic community of five monospecific AMF isolates originally obtained from a single field site in Switzerland (Jansa et al., 2002) was used to inoculate the soils. The AMF isolates originated from the same field site from which the acid soil was collected for the experiment described here. The AMF inocula were produced in open pot cultures with leek (*Allium porrum* L.) as a host plant for more than 2 years prior to setting up the pot experiment described here. Pots for production of the AMF inocula were filled with substrate composed of 10% (by volume) of sterilized soil, 45% autoclaved sand and 45% autoclaved zeolite ^1^ (1–2.5 mm grain size). For inoculum production of *Gigaspora* and *Racocetra*, we used substrate mixture containing the acid soil described above, and for production of *Funneliformis, Claroideoglomus*, and *Rhizophagus*, we used substrate with the alkaline soil (see above). Spore density in the inoculum production pots ranged from about 5 spores per gram for *Racocetra* to more than 250 spores per gram for *Rhizophagus*. Based on this rough estimate of infective propagule density and previous experience on competition between the AMF isolates (Thonar et al., 2014), we mixed the different monospecific inocula as follows: *Racocetra pellucida* BEG^2^ 153–1,800 g, *Gigaspora margarita* BEG 152–1,800 g, *Claroideoglomus claroideum* BEG 155–1,800 g, *Rhizophagus irregularis* BEG 158–900 g, and *Funneliformis mosseae* BEG 161–300 g. Half of the mix (3.3 kg) was autoclaved at 121°C for 30 min to create non-mycorrhizal inoculum, whereas the other half (3.3 kg) was used alive as mycorrhizal inoculum. One hundred grams of the inocula (either autoclaved or not, containing the substrate from previous pot cultures, fragments of leek roots from the previous pot culture, cut to <1 cm length, AMF spores and hyphae as well as other microbes) were administered to each pot.

### Plant, Soil, and AMF Analyses

Upon harvest, the shoots of the plants were cut at the substrate level, weighed fresh, dried at 65°C for 72 h and then, their dry weights were recorded. The roots were shaken off loosely adhering soil, cleaned under tap water, blotted against paper towel and fresh weight of the entire root system per pot was recorded. Thereafter, roots from each pot were cut to approximately 1-cm long fragments, mixed and a part of the roots (between 0.2 and 4 g fresh weight, exact values recorded) taken for staining and microscopy assessment of root colonization by AMF structures. The remaining roots were weighed fresh once again, then dried at 65°C for 72 h and dry weights recorded. Dried root and shoot samples were then pulverized using an oscillatory ball mill (MM200, Retsch, Haan, Germany). In those samples, concentration and isotopic composition of N were assessed using elemental analyzer (Flash EA 2000) coupled with an isotope-ratio mass-spectrometer (Delta V Advantage, Thermo Fisher Scientific, Waltham, MA, United States). The concentration of P in the biomass samples was assessed by malachite green method (Ohno and Zibilske, 1991), following incineration of the samples at 550°C as described previously (Slavíková et al., 2017). DNA from the roots was extracted using the glassmilk method (Gryndler et al., 2014), employing the internal DNA standard to check for presence of PCR inhibitors and to estimate DNA losses during the extraction (Thonar et al., 2012). DNA extraction from the soil samples (separately for the rooted soil and for the root-free patch) was carried out using the NucleoSpin^®^ Soil DNA extraction kit (Macherey-Nagel, Düren, Germany), employing SL1 lysis buffer and extraction enhancer SX, according to manufacturer’s recommendations. Taxon-specific primers and hydrolysis (TaqMan) probes targeting taxon-specific sequence motifs in the nuclear large ribosomal subunit RNA gene were used to quantify the abundance of the five AMF species administered with the AMF inoculum, in the roots of the experimental plants as well as in the soil samples. The analyses were carried out according to the quantitative real-time PCR protocol and cycling conditions described previously (Thonar et al., 2012), using StepOnePlus real-time cycler (Applied Biosystems), 20 μl reaction format (including 2 μl DNA template), Soil Biodyne chemistry (5x HOT FIREPol^®^ Probe qPCR Mix Plus with ROX) and amplicons of large ribosomal subunit RNA gene of the respective AMF taxa to calibrate the analyses. Primers and TaqMan probes were synthesized and HPLC purified in Generi Biotech (Hradec Králové, Czechia).

Roots for microscopy assessment of the colonization by the different AMF structures were processed as described previously (Püschel et al., 2016). Briefly, the samples were stored temporarily in 50% ethanol and then stained using the modified method of Koske and Gemma (1989): The roots were first macerated in 10% KOH (60 min at 90°C, followed by 25 min at room temperature), then washed with tap water, neutralized in 2% lactic acid (30 min at 90°C), and stained with 0.05% Trypan blue in LG (lactic acid–glycerol–water, 1:1:1, v:v:v) for 30 min at 90°C followed by overnight incubation in LG at room temperature. The next day, the roots were washed with tap water and further stored in LG. Colonization of the roots by AMF was quantified under a dissecting microscope at 100× magnification following the method of McGonigle et al. (1990). One hundred root intersections were observed per root sample through the eyepiece grid while recording separately the occurrence of AM fungal hyphae, arbuscules, and vesicles in each root intersection.

All different soils amended or not with the different biochars, and the biochars themselves, were analyzed for their physico-chemical properties. Specifically, we measured pH in 1:2.5 (*w:v*) slurry, and the total and water-extractable P concentrations as well as immediately plant-available P concentrations as per the isotope exchange kinetics (Frossard and Sinaj, 1998). For total P concentration assessment, soil samples (0.5 g) were first incinerated at 550°C for 12 h, extracted with 1 ml boiling HNO_3_, made up to 50 ml with ultrapure water, and the P concentrations in the extracts was measured with malachite green method (Ohno and Zibilske, 1991). Water-extractable P concentrations in the soils were assessed in samples shaken with water (1:10, *w:v*) for 18 h, filtered through 0.22-μm membrane filter and the P concentrations measured with the malachite green method as above. The C and N concentrations in the soils and the isotopic composition of these two elements were assessed using elemental analyzer coupled with isotope ratio mass spectrometer as above. Acidic soil extracts used previously for estimation of total P were subsequently used for quantification of concentrations of selected metals and potentially toxic trace elements by either inductively coupled plasma sector field mass spectrometry (ICPSFMS, Element 2, Thermo Fisher Scientific, United States) or inductively coupled plasma optical emission spectrometry (ICPOES, Agilent 5100 SVDV, United States) as appropriate (see Supplementary Table S1 for details).

### Calculations and Statistics

Total dry weight of roots per pot was calculated from the ratio of dry-to-fresh weight of the root aliquot subjected to drying × fresh weight of the entire root system in the respective pot. The P and N contents of the plants were calculated from the concentrations of the respective elements in shoot and root biomass and the biomass of the shoots and roots in the individual pots, respectively, and then summed together. Mycorrhizal growth, P uptake, and N uptake responses for the pots inoculated with living AMF inoculum were calculated as natural logarithm of a ratio of plant biomass, P or N content of the plants, respectively, in the individual pots inoculated with living AMF inoculum, and the average of the respective non-mycorrhizal control treatment. Measured abundances of the different AMF taxa in the root and soil samples (gene copies per unit weight of the samples) were corrected for recovery of the internal DNA standard per each individual sample as described previously (Thonar et al., 2012). Preferential allocation of the biomass of each AMF taxon to the root-free compartment (RFC) amended with organic N source as compared to the rooted soil was calculated for each pot as follows:

$$Hyphal\;allocation\;index\;=\;\ln\left(\frac{abundance\;of\;AMF\;taxon\;in\;RFC\;+1}{abundance\;of\;AMF\;taxon\;in\;rooted\;soil+1}\right),$$

where both of the abundances are given as gene copies of the nuclear large ribosomal subunit of the specific AMF taxon per gram of the respective soil sample.

Rates of transfer of ^15^N from organic fertilizer to the plant were calculated as described previously (Bukovská et al., 2018). The data were analyzed using one-, two-, and three-way analyses of variance (ANOVA). Significant deviation of the hyphal allocation index from zero was tested by using one-sample *t*-test. Meeting assumptions of the different ANOVA analyses were confirmed by visual inspection of the residual plots. No data transformations were needed in order to fulfill the ANOVA assumptions for data analysis. Tukey’s honestly significant different tests were used to separate means at *p* < 0.05 level following significant ANOVA. The stats were calculated in Statgraphics Plus for Windows v. 3.1.

## Results

### Biochar and Soil Properties

Both native and active biochars showed low pH values between 3.5 and 4.5, high total C concentrations, comparable total organic N and low total P concentrations relative to the soils, and very different P availabilities, with immediately available P concentrations being about 10-fold higher for the active as compared to the native (untreated) biochar (Table 1). Biochar addition to the two experimental soils has not markedly changed total P concentrations in those soils, but decreased P availability particularly in the acid soil, and the pH of the alkaline soil, besides markedly increasing C concentration of the soils (Table 1).

### Plant Growth and Nutrition

Comprehensive statistics of the effects of soil matrix, inoculation with AMF, and the addition of different biochars (and of the interactions between those experimental factors) on plant biomass production and plant mineral nutrition is given in Tables 2, 3. Due to generally strong effect of soil matrix on plant growth and mineral nutrition, and also due to multiple significant interactions between the soil and the other experimental factors, we describe the results of plant biomass production and mineral nutrient (P and N) uptake separately for the acid and alkaline soils below.

**Table 2:** Results of three-way analyses of variance of plant-related parameters, showing *F*-values and associated *p*-value ranges (ns *p* ≥ 0.05, ^∗^ 0.05 > *p* ≥ 0.01, ^∗∗^ 0.01 > *p* ≥ 0.001, ^∗∗∗^ 0.001 > *p*) for individual experimental factors and their combinations (conc., concentration).

Parameter	Soil (A)	Mycorrhiza (B)	Biochar (C)	A × B	A × C	B × C	A × B × C
DW plants^1^	136.2^∗∗∗^	60.9^∗∗∗^	103.0^∗∗∗^	6.2^∗^	67.3^∗∗∗^	3.4^∗^	16.2^∗∗∗^
P content plants^2^	99.0^∗∗∗^	119.1^∗∗∗^	100.8^∗∗∗^	4.2^∗^	43.5^∗∗∗^	0.9 ns	17.5^∗∗∗^
N content plants^3^	141.0^∗∗∗^	98.6^∗∗∗^	82.5^∗∗∗^	3.2 ns	48.0^∗∗∗^	2.4 ns	9.2^∗∗∗^
P conc.^4^ shoots	1.3 ns	211.8^∗∗∗^	15.7^∗∗∗^	9.4^∗∗^	1.4 ns	6.1^∗∗^	11.2^∗∗∗^
P conc.^4^ roots	0.03 ns	434.8^∗∗∗^	23.0^∗∗∗^	16.2^∗∗∗^	2.9 ns	3.1 ns	9.8^∗∗∗^
N conc.^5^ shoots	1.7 ns	4.6^∗^	2.4 ns	0.6 ns	22.6^∗∗∗^	4.5^∗^	17.9^∗∗∗^
N conc.^5^ roots	0.2 ns	0.03 ns	9.5^∗∗∗^	0.2 ns	21.8^∗∗∗^	0.1 ns	9.2^∗∗∗^
^15^N transport from RFC to plant^6^	9.2^∗∗^	129.8^∗∗∗^	47.6^∗∗∗^	7.7^∗∗^	15.1^∗∗∗^	0.5 ns	2.9 ns

*^*1*^ Dry weight (DW) of plants, roots and shoots combined, g/pot.^*2*^ Amount of phosphorus (P) contained in the plant biomass, roots and shoots combined, mg/pot.^*3*^ Amount of nitrogen (N) contained in the plant biomass, roots and shoots combined, mg/pot.^*4*^ Concentration of phosphorus (P) in the shoots or roots, mg/g.^*5*^ Concentration of nitrogen (N) in the shoots or roots, mg/g.^*6*^ Transfer of ^*15*^N isotope from the organic amendment placed in the root-free compartment (RFC) to the plant biomass (% of added^*15*^N)*.

**Table 3:** Results of two-way analyses of variance of mycorrhiza-related parameters, showing *F*-values and associated *p*-value ranges (ns *p* ≥ 0.05, ^∗^ 0.05 > *p* ≥ 0.01,^∗∗∗^ 0.001 > *p*) for individual experimental factors and their combination.

Parameter	Soil (A)	Biochar (B)	A × B
Mycorrhizal growth response	50.5^∗∗∗^	12.3^∗∗∗^	31.2^∗∗∗^
Mycorrhizal P uptake response	93.4^∗∗∗^	40.1^∗∗∗^	61.3^∗∗∗^
Mycorrhizal N uptake response	63.7^∗∗∗^	19.8^∗∗∗^	17.4^∗∗∗^
H%^1^	2.5 ns	0.6 ns	0.3 ns
A%^2^	0.6 ns	1.9 ns	0.1 ns
V%^3^	0.2 ns	0.4 ns	0.4 ns
*Claroideoglomus* abundance (roots)^4^	7.4^∗^	1.5 ns	1.5 ns
*Rhizophagus* abundance (roots)^4^	0.8 ns	0.4 ns	0.3 ns
*Funneliformis* abundance (roots)^4^	0.2 ns	0.3 ns	1.0 ns
*Racocetra* abundance (roots)^4^	n.a. – no positive detection		
*Gigaspora* abundance (roots)^4^	1.3 ns	1.3 ns	1.3 ns
*Claroideoglomus* abundance (soil)^4^	8.5^∗∗^	2.0 ns	3.7^∗^
*Rhizophagus* abundance (soil)^4^	0.6 ns	0.8 ns	0.1 ns
*Funneliformis* abundance (soil)^4^	6.3^∗^	2.1 ns	2.3 ns
*Racocetra* abundance (soil)^4^	2.2 ns	1.2 ns	1.2 ns
*Gigaspora* abundance (soil)^4^	2.8 ns	1.2 ns	1.1 ns
*Claroideoglomus* abundance (RFC)^4^	7.6^∗^	3.1 ns	3.5^∗^
*Rhizophagus* abundance (RFC)^4^	3.7 ns	0.5 ns	1.9 ns
*Funneliformis* abundance (RFC)^4^	0.5 ns	5.6^∗∗^	0.0 ns
*Racocetra* abundance (RFC)^4^	0.6 ns	0.9 ns	0.3 ns
*Gigaspora* abundance (RFC)^4^	0.1 ns	1.1 ns	1.6 ns

*Only mycorrhizal pots (i.e., the pots inoculated with living AMF inoculum) have been considered for the analyses presented above.^*1*^ Fraction of root length colonized by mycorrhizal hyphae (% root length).^*2*^ Fraction of root length colonized by arbuscules (% root length).^*3*^ Fraction of root length colonized by vesicles (% root length).^*4*^ Abundance of *Claroideoglomus claroideum/Rhizophagus irregularis/Funneliformis mosseae/Racocetra pellucida/Gigaspora margarita* in roots/rooted soil (soil)/root-free compartment (RFC), as assessed by quantitative real-time PCR (qPCR) using previously described primers and TaqMan probes (Thonar et al., 2012)*.

**FIGURE 2:**
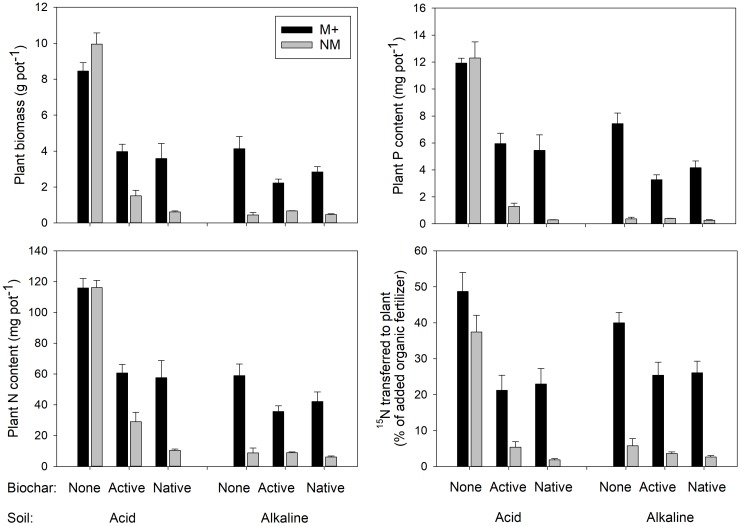
Biomass, phosphorus (P), and nitrogen (N) contents of the *Andropogon gerardii* plants and the rates of transfer of ^15^N-labeled N from the organic fertilizer (plant litter) provided in a root-free compartment to the plants. Plants were grown in either of two different soils amended or not with differently treated biochar. Black bars stand for mycorrhizal (M+) plants, gray bars for the non-mycorrhizal (NM) control treatment. Mean values +SE of means are shown (*n* = 5).

**FIGURE 3:**
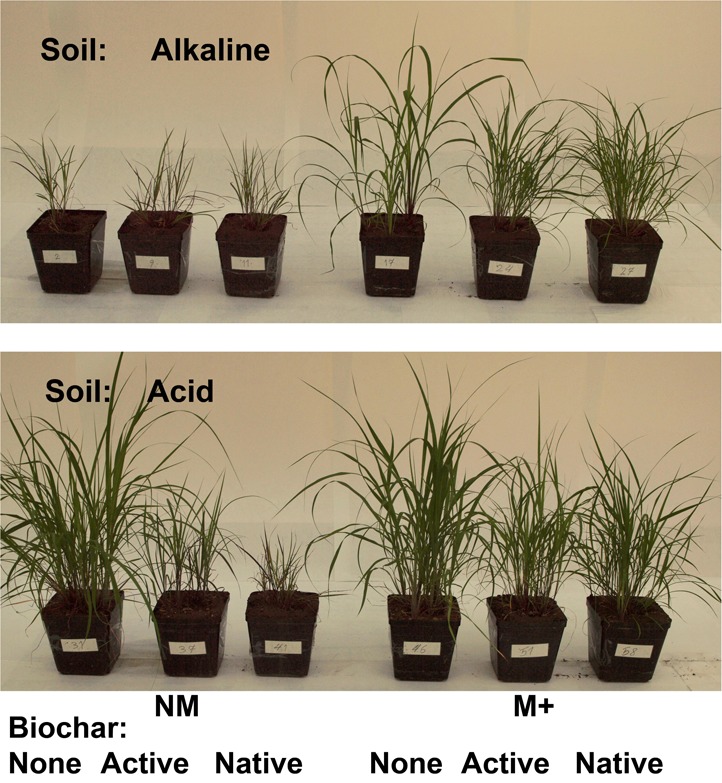
Appearance of the pots and plants shortly before harvest. The photo show mycorrhizal (M+) and non-mycorrhizal (NM) treatments in two different soils, added or not with native or active biochar.

**FIGURE 4:**
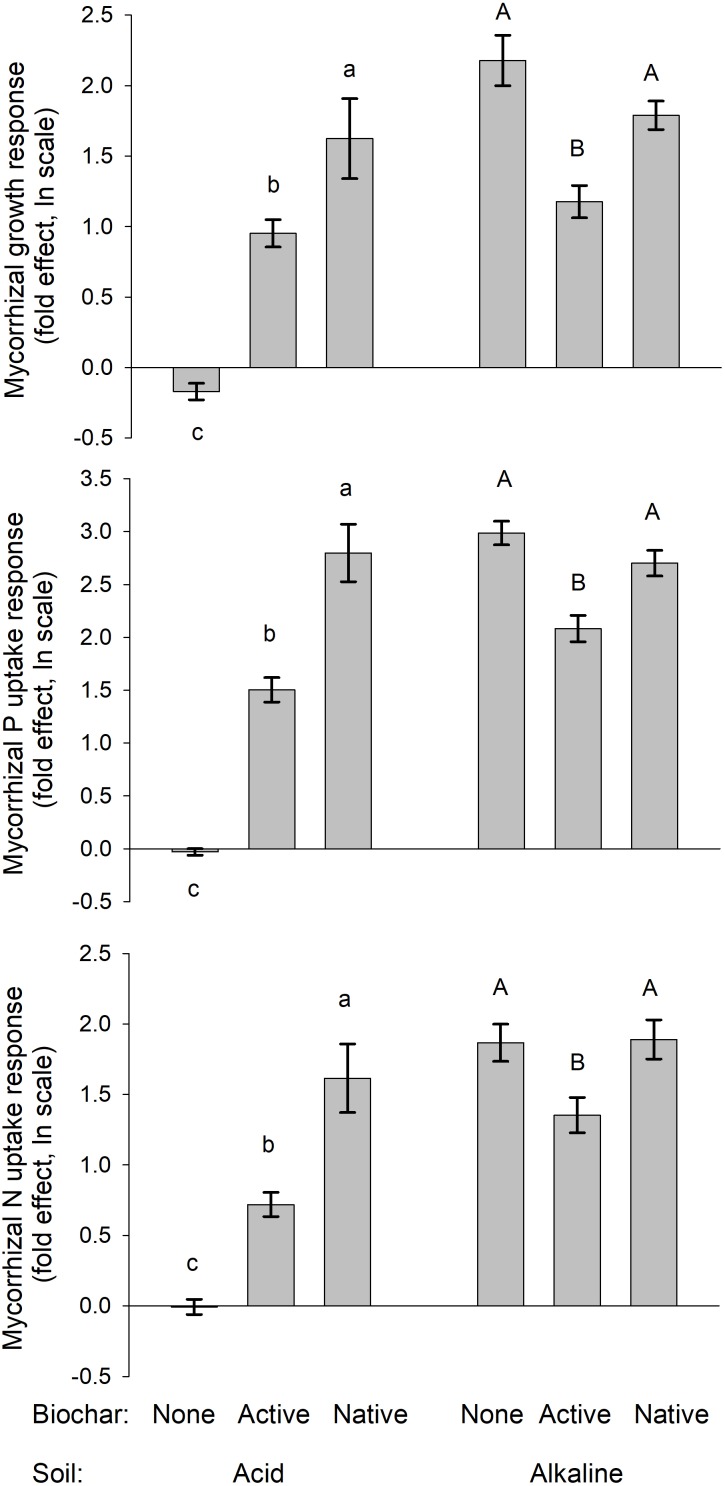
Mycorrhizal growth-, P uptake-, and N uptake-responses of the mycorrhizal plants grown in different soils and amended or not with differently treated biochar. Mean values ± SE of means are shown (*n* = 5). Different lowercase letters indicate statistically significantly different treatments within the acid soil treatment group, whereas different uppercase letters indicate different treatments within the alkaline soil treatment group.

Both the biomass production as well as uptake of P and N by the plants were strongly suppressed by biochar addition in the ***acid soil***, particularly in absence of living AMF (see Figure 2 for graphs and Figure 3 for photos). Noteworthy, the production of biomass as well as uptake of both P and N by the non-mycorrhizal plants was always lower with the native as compared to active biochar (*p* < 0.05 in all three cases). This biochar-induced suppression was to a great extent (although not fully) mitigated by AMF inoculation of the acid soil, with the differences between the native and active biochar treatments vanishing for the mycorrhizal plants (Figure 2). This pattern of effects resulted in the mycorrhizal growth- and nutrient uptake-responses in the acid soil being highest for the native biochar treatment, intermediate for the active biochar treatment and none (to slightly negative, *p* = 0.04 for a *t*-test addressing the difference of mycorrhizal growth response from zero) for the plants growing in acid soil without any biochar addition (Figure 4).

Growth and nutrition of the experimental plants in the ***alkaline soil*** was mainly affected by AMF inoculation. Non-mycorrhizal plants were generally stunted in alkaline soil amended or not with the different biochars, with no significant differences between the different biochar treatments (Figure 2, see also Figure 3 for photos). When mycorrhizal, the plants produced the largest biomass in and took the highest amounts of N and P from alkaline soil without biochar addition, whereas addition of the soil with active biochar always resulted in poorer performance (be it growth or mineral nutrition) of the plants as compared to those growing in absence of any biochar (*p* < 0.05 for all three cases). This resulted in the mycorrhizal growth- and nutrient uptake-responses being high and not significantly different from each other for plants growing in soil without biochar or with native biochar, whereas the responses were always smaller for the active biochar treatment in the alkaline soil (Figure 4).

### Transfer of ^15^N From Organic Fertilizer to Plants

Transfer of ^15^N from the organic fertilizer administered within the RFC to the plant was affected mainly by the inoculation with AMF, with only a minor contribution of the other experimental factors (Table 2). Mycorrhizal plants showed systematically higher rates of ^15^N uptake from the organic fertilizer as compared to the non-mycorrhizal plants (Figure 2 and Table 2 for the stats). The second most influential factor affecting ^15^N uptake by plants from the organic fertilizer was biochar addition. Plants growing in soils without biochar addition showed generally higher rates of ^15^N transfer than those growing in soils added with one or the other biochar (Figure 2). There was also a significant effect of soil, as well as interactions of soil × mycorrhizal inoculation and soil × biochar amendment, but the share of explained variability due to these other factors or their interactions was much smaller than the variability explained by the first two single factors and thus the interactions are not further elaborated here (but see Table 2 for the complete stats).

### Mycorrhizal Colonization of Roots and Soils

**FIGURE 5:**
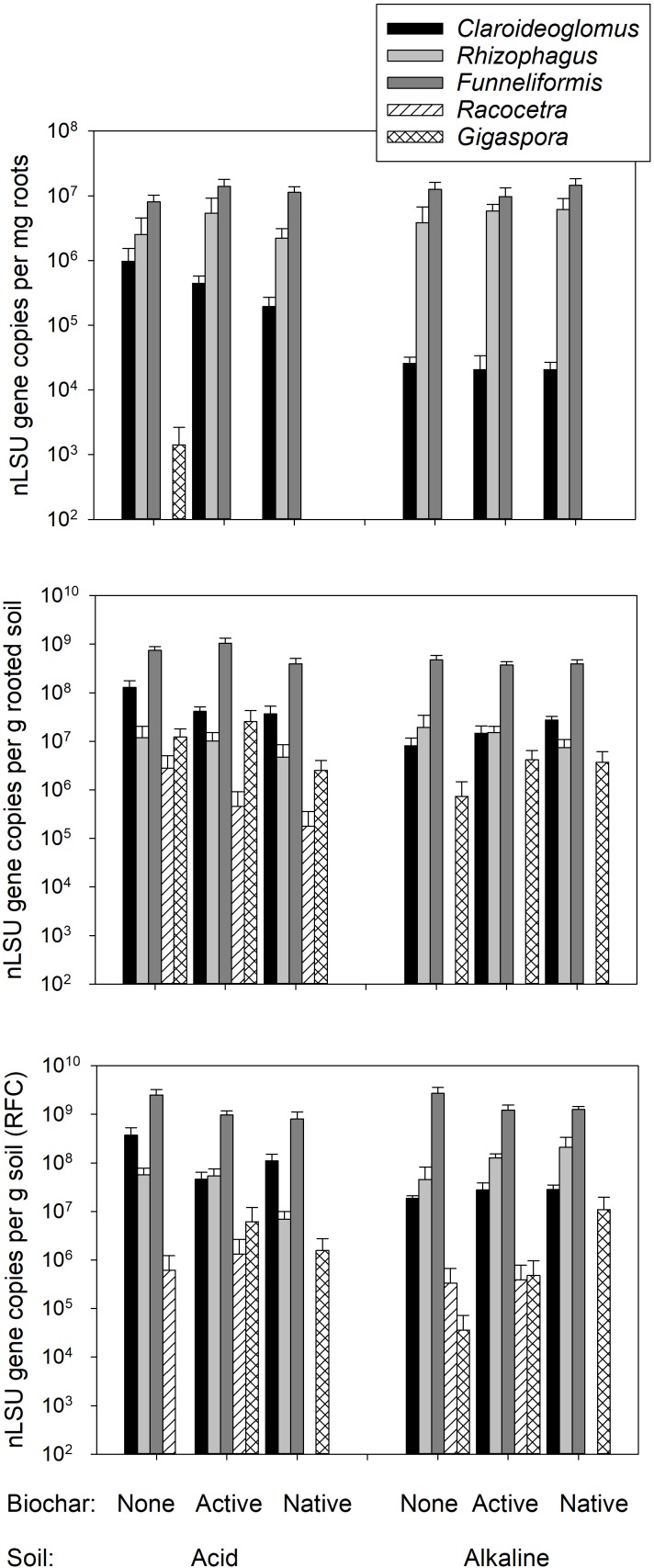
Abundance of the different arbuscular mycorrhizal fungal (AMF) taxa in the roots of plants inoculated with living mycorrhizal inoculum, rooted soil and the root free compartment (RFC), as assessed by quantitative real-time PCR targeting taxon-specific motifs in the nuclear large ribosomal subunit (nLSU) RNA gene. Mean values +SE of means are shown on a logarithmic scale (*n* = 5).

The extent of root length colonized by mycorrhizal fungi was not affected in the plants provided with the living AMF inoculum by any of the experimental factors for either of the individual structures recorded microscopically (grand mean ± SE across all mycorrhizal plants: hyphae 34.8 ± 2.9 %, arbuscules 20.4 ± 2.0%, and vesicles 3.3 ± 0.8%, see Table 3 for the stats). No AMF structures were observed in the roots of plants inoculated with autoclaved AMF inoculum (see data in Supplementary Table S1). Abundances of individual AMF taxa in the roots of plants provided with the living AMF inoculum was not affected by any of the experimental factor except *Claroideoglomus* sp., which was significantly less abundant in the roots of plants growing in alkaline as compared to the acid soil (Figure 5 and Table 3). *Racocetra* was not detected in any root sample, and only trace amounts of *Gigaspora* were detected in the roots from two pots inoculated with the living AMF inoculum and filled with acid soil not amended with any biochar (see Supplementary Table S1 for details).

Systematically higher abundances of *Claroideoglomus* and *Funneliformis* were recorded in the acid as compared to the alkaline soil (Figure 5 and Table 3). The abundance of *Claroideoglomus* in the rooted soil was further modulated by biochar addition so that the native biochar amendment stimulated (*p* < 0.05) its abundance in alkaline as compared to biochar-free alkaline soil, whereas its abundance tended (*p* < 0.1) to be lower in biochar-amended acid soils than in the acid soil without biochar, resulting in significant interaction between the two (i.e., soil and biochar) experimental factors (Table 3). A similar response pattern for *Claroideoglomus* abundance was also observed in the soil collected from the RFC (Table 3 and Figure 5). In the RFC, we also observed a strong and systematic suppression of *Funneliformis* development by both biochar amendments, regardless of the soil matrix (Table 3), with the values in biochar-amended RFC reaching only about one third of the values observed in the biochar-free soils (see Figure 5 and Supplementary Table S1 for details).

For the three dominant AMF taxa (i.e., *Funneliformis, Rhizophagus*, and *Claroideoglomus*), we observed significantly greater hyphal allocation to the organic N-amended RFC than to the rooted soil compartment (*p* < 0.05 in all three cases), whereas no preferential hyphal allocation to the RFC was observed for *Gigaspora* or *Racocetra* (see Supplementary Table S1 for data, analyses not shown). Besides, preferential hyphal allocation to the RFC was greater in alkaline soil than in the acid soil for *Funneliformis* (*p* < 0.05). Hyphal allocation to the RFC was never affected by any biochar application for any of the AMF taxa included in this study (analyses not shown).

## Discussion

Establishment of arbuscular mycorrhizal symbiosis was obviously very important for *Andropogon* growth and nutrition in the alkaline soil (see also previous research: Bukovská et al., 2018; Gryndler et al., 2018), whereas the performance of the experimental plants in the acid soil (without biochar) was not significantly improved by the AMF inoculation (Figure 2). Addition of biochar to either of the soils was generally suppressing plant growth and nutrition as compared to the respective soil treatments without biochar. In the acid soil, mycorrhizal symbiosis significantly (though not fully) counteracted biochar-induced growth and nutrient uptake suppressions. In the alkaline soil, the growth of non-mycorrhizal plants was so stunted that no detrimental effects of biochar (as compared to the treatment without biochar) were detectable in the non-mycorrhizal control treatment. Yet, the mycorrhizal plants growing in biochar-amended alkaline soil grew smaller and took up less nutrients than those growing in the soil without biochar, indicating negative effect of biochar on our experimental plants, which obviously could not fully be restored by the symbiosis with the AMF. Interestingly, the active biochar caused systematically lower mycorrhizal growth and nutritional responses than the native biochar in both of the soils included in this study (Figure 4). These results were surprising and partly cross to our expectations of neutral to positive effects of biochar on plant growth and/or mineral nutrition, in concert with the earlier literature reports (Jeffery et al., 2011; Spokas et al., 2012; Biederman and Harpole, 2013). Further, we expected synergistic and positive effects of both biochar and AMF on the plants, whereas the actual outcome of the interaction turned to be very much dependent on the soil matrix. Our results indicate that biochar either directly intoxicated the plants and/or interfered with their nutrient uptake or that it caused changes in the soil microbiome with detrimental consequences for the plant nutrition and/or growth. These different scenarios and the fact that the detrimental effect of biochar could at least partly be counteracted by the AMF, deserve specific attention here.

### Possible Biochar Phytotoxicity

Freshly prepared biochar could be toxic to the plants due to presence of a variety of tars and oily substances including PAHs (Dutta et al., 2017; Liu et al., 2017; Zhu et al., 2017, and references therein). By using aged biochar that spent decades in the forest soil prior to the experiment described here, being exposed to temperature fluctuations, biological activity and percolating rainwater throughout the years, we expected to eliminate presence of such toxic and partly volatile compounds in our experiment – although, admittedly, we did not measure presence of such compounds directly nor did we carry out any standardized toxicity biotest with our biochars. Still another option would be that the biochar could have, over the years, absorbed significant amounts of toxic elements or other environmental pollutants. Then the biochars could poison our experimental plants in the pots. However, given no elevated toxic element concentrations in our biochars were detected (see Supplementary Table S1 for data), and also because we did not observe any specific toxicity symptoms such as leaf discolorations or stripes (personal observations), direct toxicity of the biochars to our experimental plants is rather an unlikely scenario.

### Biochar Interference With Plant Nutrition

Untreated historic biochar applied to acid soil decreased the growth and N uptake of non-mycorrhizal *Andropogon* plants more than 10-fold. Furthermore, the P uptake of the non-mycorrhizal plants decreased more than 30-fold due to application of the untreated biochar into acid soil, whereas the negative effects of active biochar on non-mycorrhizal plants in the acid soil were much less prominent (Figure 2). We interpret this as extraordinary capacity of the untreated historic biochar to decrease P availability in the acid soil (although the measured decrease in immediately available P pool due to untreated biochar application to acid soil was “only” up to sixfold, Table 1). Mycorrhizal plants in both acid and alkaline soils were suppressed by about 50% in their growth and mineral (P and N) nutrition as compared to the mycorrhizal plants in the respective soils without biochar (Figure 2). In alkaline soils, the suppression of plant growth and mineral uptake tended to be greater in soils added with active as compared to untreated biochar. This may be a result of higher P availability in the soil due to application of active as compared to untreated biochar (Table 1 and Figure 3), in line with resource stoichiometry framework (Johnson et al., 2010, 2015) which predicts that increasing P availability in soil loosens tight mutualism between the plant and the AMF. The plants, in consequence, become more reliant on the direct (root) P uptake pathway upon active as compared to untreated biochar application, which may however not be able to fully compensate for particularly effective P acquisition via the indirect (mycorrhizal) P uptake pathway (see Smith and Smith, 2012, for further discussion).

**FIGURE 6:**
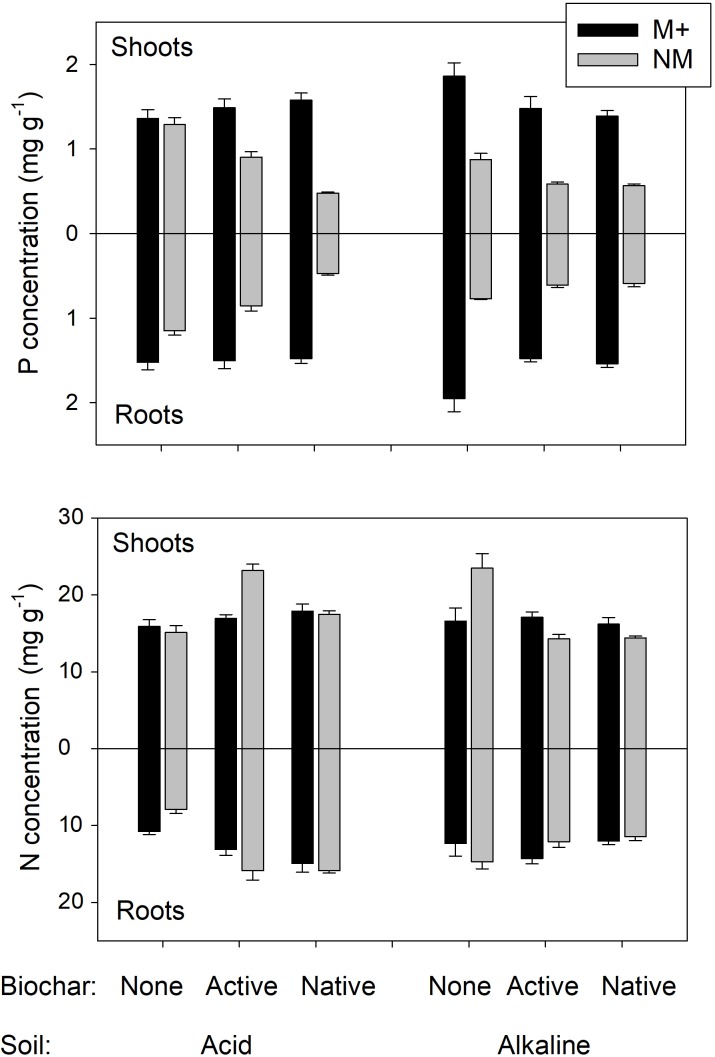
Phosphorus (P) and nitrogen (N) concentrations in shoots and roots of experimental plants. Plants were grown in either of two different soils amended or not with differently treated biochar. Black bars stand for mycorrhizal (M+) plants, gray bars for the non-mycorrhizal (NM) control treatment. Mean values +SE of means are shown (*n* = 5).

Collectively, the above results indicate that growth of the plants in our experimental system was primarily limited by P availability in the differently treated soils. This is because both P concentrations (Figure 6) and also P contents of plants (Figure 2) were usually dramatically increased by presence of AMF, whereas biochar generally caused a decrease in both P concentrations and P contents of the plants (consistent with its effect on decreasing available P in soil, see Table 1 for P availability data and Table 2 for the plant experiment stats). The effects of experimental treatments on N concentration in the plants (that would be suggestive of N limitation) have been much milder as compared to the effects on P concentrations/contents (Figure 6 and Table 2). Importantly, the N:P ratio sharply increased with decreasing plant biomass, being around 10 for large plants (3 g total dry biomass per pot and higher) and rocketing up to 90 for the smallest plants (see Supplementary Table S1 for data). This further suggests that it was the P, and not the N, which was the primary limitation of the growth, and also explains why mycorrhiza was such a prominent factor of plant growth/nutrition in our experimental system – because of its well-recognized role in plant P nutrition, namely supplying P from soil to the host plant via the mycorrhizal (indirect) P uptake pathway (Smith et al., 2004; Johnson et al., 2015). Such a pathway is highly effective under low P availability in soil, but may become an energetic burden for the plant under high P availabilities, resulting in down-regulation of the mycorrhizal colonization of roots and also in declining mycorrhizal benefits upon elevated P concentration in soil (Konvalinková et al., 2017 and references therein).

There are several possible options to explain how biochar does lower the P availability in the soil – both for the roots and also for the AMF hyphae. This could be achieved either directly, e.g., by adsorbing orthophosphate onto inner surfaces of biochar particles (see Figure 1 for photos) that could not easily be accessed by roots and/or the hyphae due to small pore sizes. Further, it could promote soil aggregation and/or stabilize Al/Fe/Ca complexes that could either irreversibly bind inorganic orthophosphate or make it spatially inaccessible to (hidden from) the roots and/or hyphae (Dai et al., 2017; Zhang et al., 2017; Borno et al., 2018). Biochar could also bind/inactivate root or hyphal exudates such as organic acid responsible for increasing P availability in the immediate vicinity of the roots/hyphae (Lehmann et al., 2011; Spokas et al., 2011; Ameloot et al., 2013; Sun et al., 2016; Koide, 2017). The biochar could, in addition, also suppress microbial nutrient cycling (Prommer et al., 2014) which could have consequences for both P and N availabilities to the roots and/or to the AMF hyphae. Still another scenario would be that some microbes would directly feed on the biochar C (Zhu et al., 2017), and in consequence immobilizing inorganic nutrients such as P and/or N from the soil solution in their biomass – such a scenario is however rather unlikely for aged biochar that has previously been exposed to microbial degradation for a couple of decades such as in our case.

Whereas mycorrhizal symbiosis certainly has improved P nutrition of our plants (see above), it also seems to have positively affected plant N acquisition from organic N source labeled with ^15^N and supplied in the RFC. Noteworthy, the ^15^N transfer from the organic fertilizer to the plants correlated significantly with size of the plants (*R*^2^ = 68.9%, *p* < 0.001). This may mean, on one hand, that ^15^N was taken up mainly passively with the water mass flow (which would be much higher for larger than for smaller plants) or that it was taken up actively to AMF hyphae and then transported to the plants, because mycorrhizal plants were generally larger that their non-mycorrhizal counterparts, particularly in the biochar-amended soils. This latter notion is indirectly supported by the fact that the ratio of the amount of ^15^N transferred from the RFC to the plants was nearly twice as high for mycorrhizal as compared to the non-mycorrhizal plants (*t*-test *p* = 0.024) growing in acid soil without biochar (size of the plants in those two treatments was namely well comparable, Figure 2). This is also consistent with previous literature providing experimental evidence for active N transport from soil to plant via AMF hyphae (Mäder et al., 2000; Hodge et al., 2001; Hodge and Fitter, 2010; Bukovská et al., 2018). What needs further research, however, is whether and how the AMF promoted organic N mineralization. There is quite some controversy on this topic in the literature; an obvious problem being the fact that AMF are thought to be completely dependent on other microbes to release mineral N from organic N sources. However, some other reports also show that AMF may also suppress microbial activity in the soil through effectively mining the mineral N and P from the soil solution, or through direct or indirect allelopathy (Herman et al., 2012; Nuccio et al., 2013; Gui et al., 2017; Bukovská et al., 2018; Koide and Fernandez, 2018). Further research in this direction is thus certainly warranted.

### Possible Changes of Soil Microbiome Due to Biochar Application

Biochar application can exert significant changes on soil microbial communities (Zhu et al., 2017; Zhang et al., 2018). Theoretically, biochar could stimulate specific plant-pathogenic microorganisms that would negatively affect *Andropogon* performance in our pot experiment and this apparent negative effect could be counteracted by the AMF because the AMF have previously been reported to play a role in plant tolerance to pathogens (e.g., Newsham et al., 1995). The native biochar could also carry with it some living microorganisms that could affect the plants/AMF in the pots – whereas the active charcoal is unlikely to cause any significant biological inputs, in contrast. However, thorough investigation of the microbial (e.g., prokaryotic) communities in the different soil treatment was beyond the scope of the research described here. Therefore, it remains a speculation whether native biochar introduced any specific plant pathogens to our experimental system or whether the biochar amendment to our pots consistently stimulated any pathogenic microbes from the pool of microbes already present in the pots.

Our analyses concentrated on the abundance of AMF taxa inoculated into the pots and on possible AMF inputs (contaminations) with the untreated biochar, recovered from the forest floor. And there we saw surprisingly little effect of the biochar on both the extent of root colonization by the inoculant AMF (Table 3) and the abundance of the individual AMF taxa in the roots and in the soil (Table 3 and Figure 5). These results are in line with the field observations showing no major effect of biochar on AMF (e.g., Camenzind et al., 2018). Besides, we obtained no evidence for any significant AMF load with the native biochar as the plants growing in soil with untreated biochar and not inoculated with living AMF all remained non-mycorrhizal (see Supplementary Table S1 for data).

Whereas there was no strong effect of biochar on most of the AMF taxa and the extent of root colonization by AMF structures in our pots experiment, both *Claroideoglomus* and *Funneliformis* showed some preference for the acid soil, which was actually their home environment – possibly because of the soil pH (Jansa et al., 2014). This latter notion is further supported by the fact that *Claroideoglomus* was differentially affected by biochar application into the different soils, co-incident with pH shifts induced by the biochar application (compare Table 1 and Figure 5). The reason why preferential hyphal allocation of *Funneliformis* to RFC was greater in alkaline than in the acid soil remains unclear, however – the hyphae could be attracted either by free N or other cues, but we have no unequivocal mechanistic explanation for the observed effect at this stage of research.

A notable and unique observation was that biochar specifically and systematically suppressed hyphal development of *Funneliformis* in the RFC, though not necessarily in the rooted soil (Figure 5). This may have something to do with biochar changing porosity of the soils as it has previously been shown that AMF hyphal growth could indeed be affected by soil porosity (Drew et al., 2003; Martin et al., 2012). Why *Funneliformis* and not the other AMF taxa reacted to biochar remains unclear, though. Another explanation is that the combination of biochar with organic fertilizer in the RFC was a particularly unsuitable environment for *Funneliformis* (but see above for the discussion on the differential hyphal allocation of *Funneliformis* to RFC in the different soils). Since we did not include RFC without organic fertilizers nor we did compensate for N inputs in the rooted soil, elucidating possible specific interactions between *Funneliformis* hyphal networks, organic N fertilizer and biochar additions, and distance from the roots, would require lot of additional research efforts.

### Caveats

There were a few uncontrolled factors in our experiment that could have partly biased the results and/or their interpretation:

First, all pots were sprayed with an insecticide in order to prevent uncontrolled damage of the plants by insects. Whereas spraying all pots (and not just their selection) hopefully eliminated a systematic bias of any of the experimental factors tested here, pesticide inputs could have stimulated or suppressed the AMF, for example. This was not tested here for obvious reasons (because it would require a whole new experiment and selective application of the pesticide on some pots and not on others). Yet the insecticides are usually exerting only a mild effect on the AMF, in contrast to herbicides or fungicides (Jansa et al., 2006).

Second, autoclaving of mycorrhizal inoculum for addition into the non-mycorrhizal control treatment could have affected nutrient (e.g., P) availability or other physico-chemical soil properties as reported before (e.g., Mahmood et al., 2014, and multiple references therein). Yet the mycorrhizal inoculum only contained 10% (by volume) of soil, the rest being intact carriers (zeolite and sand), so the effects on nutrient availability in the entire pot (containing about 1 kg of γ-rays sterilized soil each) amended with as little as 10 g autoclaved soil (and some organic matter including leek roots from the previous pot cultures) was likely negligible. Since the soil for filling the pots was not autoclaved but only γ-rays sterilized (well in advance of the experiment setup), the bias due to soil sterilization should not invalidate the results of our study (although, admittedly, it could have affected them to some limited extent).

Third, we did not compensate for differential porosity and/or volume of biochar-added soils in the biochar-unamended treatments. Given the rates of biochar amendments in our experiment were rather high and the density of the biochar being generally very low compared to soils, this is an issue that should be paid particular attention to in the future (see also Koide, 2017, for extensive discussion on this topic).

Fourth, we only used AMF originally isolated from the acid soil, so they were, strictly speaking, non-native to the alkaline soil. This could have explained some of the soil effects on the abundance of the individual AMF taxa (see above), although at least three of the AMF strains were previously cultured (“trained”) in a mixture of the alkaline soil included in this study, zeolite and sand (to produce the AMF inoculum). Admittedly, it would be interesting to directly scrutinize whether soil origin of the AMF isolates had any systematic effect on the interactions of AMF with the biochar, although this would require a whole research program to be thoroughly addressed. Particularly, the AMF native to the alkaline soil included in our study are not yet available in pure cultures for conducting pot experiments such as described in this study.

Fifth, organic fertilizer labeled with ^15^N was provided in a root-inaccessible nutrient enriched patch, with the nutrient inputs not compensated for in the entire volume of the pot. This is a specific situation, which is justified to test localized response of AMF hyphae to elevated organic nutrient inputs (e.g., Hodge et al., 2001; Bukovská et al., 2016, 2018), although different research questions would have required differently designed experimental setup.

## Conclusion and Outlook

Here, we observed strong and negative effects of aged biochar (either untreated or chemically activated) on the growth and nutrition of *Andropogon gerardii* plants in two different soils, and partial remediation of the negative effect of biochar on the plants by inoculation with synthetic AMF communities. Biochar did not strongly affect the composition of the AMF communities nor did it affect the extent of root colonization by AMF structures, although we noted some negative effect of biochar on spatial spread of soil hyphae of *Funneliformis mosseae*. We interpret our results mainly as biochar interfering with root P uptake from soil (most likely by decreasing P bioavailability in soil directly through sorption of free orthophosphate ions from soil solution or occluding soil sorption sites, responsible for exchange of P ions between soil solid phase and soil solution). This is supported by the fact that all poorly growing non-mycorrhizal plants (i.e., all those in alkaline soil and those growing in acid soil added with native biochar) invariably showed symptoms of P deficiency such as violet coloration of leaves (Figure 3). Because the AMF provide an alternative P uptake pathway to the direct (root) uptake pathway (e.g., Smith and Smith, 2012), which was likely more effective than the root uptake pathway in biochar-amended soils, the AMF could partly remediate the biochar-induced suppression of plant P nutrition and growth. Our research described here is limited only to one plant species (notably, a non-native plant species to Europe, and the experiment conducted with two European soils), on which biochar application obviously had a strongly negative impact in terms of growth and mineral nutrition. Additional experiments with other plant species, other soils and other biochars and their application rates will thus be needed to allow generalizations (or not) of the results of biochar application and mycorrhizal symbiosis interactions reported here. Particular attention should then be paid to separation of physico-chemical and biological mechanisms by combining sterilization/microbial inoculation treatments and isotopic labeling to directly trace nutrient (and possibly also C) flows in the experimental system. Further, it needs more dynamic (time-series) studies and also testing different biochar amendments in combination with organic nutrient sources as they are thought to interactively affect plant and crop performance in the field soils (Atkinson et al., 2010; Song et al., 2014; Butnan et al., 2015; Ohsowski et al., 2018).

## Author Contributions

ZP conceived and conducted the experiments and carried out the qPCR analyses on root DNA samples. MG helped with biochar activation and analyses. TK helped with the harvest and manuscript revisions. OB conducted electron microscopy and EDS microanalyses of biochar samples. JB conducted the metal and trace element analyses of soil and biochar. PB provided the ^15^N-labeled organic fertilizer and helped with the pot experiment harvest and qPCR analyses. DP prepared the soils, helped with the harvest and contributed to revisions of the manuscript. VŘ helped with the harvest and revised the manuscript. MS helped to designing the experiments and data interpretation. JJ helped to designing the experiments and wrote first draft of the manuscript. All authors contributed to revisions and approved the final version of the manuscript.

## Conflict of Interest Statement

The authors declare that the research was conducted in the absence of any commercial or financial relationships that could be construed as a potential conflict of interest.

## Footnotes

1 www.zeopol.com

2 The International Bank for the Glomeromycota, www.i-beg.eu.

## Supplementary Material

TABLE S1 Original (raw) experimental data and datalogger records of the glasshouse condition.
